# Changing trends in seroprevalence rates of transfusion-transmitted diseases among blood donors in Jordan

**DOI:** 10.1186/s12879-021-06196-3

**Published:** 2021-05-31

**Authors:** Lina Souan, Mahmoud Siag, Hala Al-Salahat, Tareq Al-Atrash, Maher A. Sughayer

**Affiliations:** Department of Pathology Laboratory Medicine, King Hussein Cancer Center, Al-Jubeiha, Amman, Jordan

**Keywords:** Donors, Transfusion-transmitted disease, Anti-HBcore antibody, Anti-HCV, Herd immunity, vaccine

## Abstract

**Background:**

Hepatitis B and C infections and transmission are a serious challenge to all healthcare systems. We studied seroprevalence rates of Transfusion Transmitted Diseases (TTD) among blood bank donors in Jordan from 2014 to 2019 as a follow-up study of our previously published work. In addition, we wanted to explore the efficacy of the mandatory vaccination of infants against hepatitis B virus (HBV) which was implemented by the Ministry of Health since 1995 for the eradication of HBV infection in Jordan.

**Methods:**

We reviewed blood bank donors’ records at King Hussein Cancer Center (KHCC) from January 1st, 2014, until December 31st, 2019. Results of seropositivity prevalence rates for HBsAg, anti-HBcore, and anti-HCV, using Enzyme-Linked ImmunoSorbent Assay (ELISA) were compared to seropositivity rates from our previously published data. In addition, our results were compared to data obtained from other blood banks in Jordan, as well as compared to published information from blood banks in neighboring countries.

**Results:**

The prevalence rates (%) of seropositive blood donors for viral hepatitis for the years 2014, 2015, 2016, 2017, 2018, and 2019, were as follows: HBsAg rates were 0.3386, 0.2108, 0.1801, 0.1898, 0.2068, and 0.2741; anti-HBcore rates were 4.1112, 3.2271, 2.9748, 2.8405, 2.6879 and 3.0986; and anti-HCV rates were 0.1129, 0.0486, 0.0548, 0.0654, 0.0782, and 0.0839, respectively. There was a significant increase in the prevalence of HBsAg, Anti-HBcore and Anti-HCV antibodies in 2019 (one sample z-score test, *p* < 0.00001).

**Conclusions:**

Prevalence rates of hepatitis B and C infections among Jordanian blood bank donors showed a steady decline between 2009 and 2017, and these rates were much lower in Jordan than in neighboring countries. However, an increase in the prevalence rates of hepatitis B and C infections among blood bank donors was documented in 2019. While the reasons for this increase are not clear yet, these findings highlight the importance of renewed efforts to increase public health awareness of HBV and implement effective measures to prevent the transmission and infection with HBV, including national vaccination programs.

## Introduction

Screening of donated blood products for infectious diseases such as hepatitis is crucial to prevent the transmission of diseases. In our previously published study on the seropositivity rates of HBsAg, anti-HBcore, and anti-HCV among healthy Blood Bank donors at King Hussein Cancer Center (KHCC), between the years 2009–2013, we found that these rates were lower in these donors relative to published data in neighboring countries [1, 2]. Moreover, none of these donors in our previous study tested positive for anti-HIV I/II, anti-HTLV I/II, or anti-TP [2].

In this study we wanted to make a six-year follow-up on our previously published work in light of the current changes in the country and region demographics. Furthermore, we explored whether the introduction of a mandatory HBV vaccine in 1995 and the gradual entry of vaccinated donors into the donor’s pool starting in 2013 may have led to a significant decrease in seropositivity rates of HBV among blood donors from 2014 to 2019. We have undertaken therefore this new study to explore the prevalence rates of seropositive donated blood for HBsAg, anti-HBcore, and anti-HCV, from the years 2014 to 2019, among KHCC blood bank donors.

In addition, we compared our data from this study with data collected from all national blood banks in Jordan; an initiative that hasn’t been done or published before. Having updated epidemiological data is important in enrolling and retaining safe blood donors, while knowing the prevalence of infectious markers in the general population aids in recognizing low-risk donor populations [3].

## Materials and methods

The clinical Immunology laboratory at King Hussein Cancer Center (KHCC) performs the screening of blood bank donor samples following the guidelines of the College of American Pathologists’ [4]. Enzyme-linked immunosorbent assay (ELISA) technique is used for the majority of the serological screening tests such as the anti-HCV, HBsAg, and anti-HBcore. As for the Jordanian national blood bank and all blood bank centers in Jordan, they use the ELISA technique as well for screening their blood bank donor-samples. Subjects:

A retrospective cross-sectional study was done on 88,565 blood and apheresis-platelet healthy donors at KHCC between January 2014 and December 2019. The donors were adults ranging in age from 18 to 60 years of age. All blood and platelet donors were either volunteers or family replacement Jordanian citizens and were interviewed according to standard international blood donor rules and regulations. Blood bank donor-samples were processed as in our previous published work [2].

Data collected from KHCC healthy donors was compared with data collected from blood banks around Jordan. Raw data from the National-Central Blood Bank at Al-Basheer hospital, National Blood Bank (NBB) branches in Amman, NBB in Zarqa, NBB in Irbid, NBB in Al Karak, Islamic Hospital Blood Bank, Jordan University blood bank, University of Science and Technology Blood bank, Jordanian Royal Medical Services blood bank were shared with us by the National-Center Blood bank. Data from all these blood banks was added together and listed under one group titled “National Blood Bank”. The total number of blood donors from all national blood banks during the same period was 1,224,933 donors.

### HBsAg screening

Screening for HBsAg was performed as in [2]. The specificity of Murex HBsAg Version 3 is estimated to be 99.97% (12,326/12330) with a 95% confidence interval of 99.92% (12,320/12330) to 99.99% (12,328/12330) by the binomial distribution [5]. The sensitivity of the Bio-Rad Monalisa HBsAg sandwich ELISA test is shown to be 100% with a specificity on a total of 9894 random blood donors from 3 different sites equals to 99.94% (9887/9893) [6]. The ARCHITECT HBsAg Qualitative II assay is designed to have a specificity of greater than 99.5% on blood donor specimens [7]. Roche HBsAg Qualitative assay test sensitivity was reported to be 100% with a specificity of 99.98% [8].

### Anti-HBcore antibody screening

Screening for Anti-HBcore antibodies was performed using Bio-Rad Monolisa Anti-HBc PLUS assay kit (Bio-Rad, Marnes-la-Coquette, France) as detailed in [2]. The diagnostic sensitivity, performed on 430 positive samples with EIA reference test for the Bio-Rad Monolisa Anti-HBc PLUS assay test is 99.53% (428/430). The specificity of the test on 5071 samples in non-selected blood bank donors is 99.9. The specificity of the test evaluated on 439 patients is 99.5% [9]. The sensitivity of the ARCHITECT Anti-HBc II assay was evaluated with a four-member panel that was standardized against reference serum from the Paul-Ehrlich-Institute (PEI). The ARCHITECT Anti- HBc II assay test is designed to show an analytical sensitivity of less than 1.0 PEI-U/mL. The panel was tested with three reagent lots. The ARCHITECT Anti-HBc II assay sensitivity ranged from 0.4 to 0.5 PEI U/mL. The ARCHITECT Anti-HBc II assay is designed to have an overall specificity of ≥99.5% on a blood donor population and ≥ 98.0% on a hospitalized or diagnostic population [10]. The Roche Anti-HBc antibody assay test sensitivity is reported to be 100% with a specificity of 99.7% [11].

### Anti-HCV antibody screening

Screening for anti-HCV antibody was performed using DIAsource anti-HCV V 4.0 (DIA-source ImmunoAssays SA, Nivelles, Belgium) ELISA kits as in [2]. Testing procedure and interpretation of the results were done according to the manufacturer’s instructions. Samples giving equivocal (gray zone) readings were confirmed by repeating the sample in duplicate using the same kit and testing it using another methodology such as the CMIA on Abbott Architect i1000 analyzer, ARCHITECT Anti-HCV assay kit (ABBOTT, Wiesbaden, Germany), After February 13, 2018, confirmatory tests were performed on Cobas e601 Roche analyzer 6000, Roche Anti-HCV antibody assay kit (Roche Diagnostics International Ltd., Switzerland) using Electrochemiluminescence Method (ECLIA) method. Repeatedly positive samples using the ELISA method were considered positive for Anti-HCV antibody.

The diagnostic sensitivity of the DIAsource ImmunoAssays SA Anti-HCV Elisa V 4.0 test was determined to be 100% with an analytical specificity of 99.8% [12]. The overall specificity for the ARCHITECT Anti-HCV assay test was 99.60% (10,361/10,403) with a 95% confidence interval of 99.45 to 99.71%. The specificity observed ranged between 99.20% (496/500) to 99.70% (1994/2000). The sensitivity was 99.10% with a 95% confidence interval of 96.77 to 99.89% [13]. As for the Roche Anti-HCV antibody assay the diagnostic sensitivity was 100%. The 95% lower confidence limit was 99.61%. The diagnostic specificity of the Elecsys Anti-HCV II assay in a group of hospitalized patients was found 99.66%. The 95% lower confidence limit (2-sided) was 99.41–99.82% [14].

### Statistical methods

We performed a descriptive data analysis of the results of blood donors’ screening tests for HBsAg, Anti-HBV core antibody, and Anti-HCV antibody, and the data are presented as counts and percentages for both KHCC and national blood banks. Comparison between the current period (2014 to 2019) with the previously reported period (2009 to 2013) was carried out using t-test to determine if there is a significant difference between the means of two groups.

Comparisons between years (2009 to 2018) in the KHCC donor screening tests (HBsAg, Anti-HBcore, and Anti-HCV) was carried out using Chi square test or Fisher exact depending on the assumption required for each test. The same was done to compare positive results of HBsAg and Anti-HCV in the KHCC and National BB donor populations according to year.

Trend was assisted and presented using Joinpoint regression program, and Annual Percent Change (APC) was reported in addition to significant critical points.

A significance criterion of *p* < =0.05 was used in the analysis. All analyses were performed using SAS version 9.4 (SAS Institute Inc., Cary, NC).

One-sample z-test was used to determine whether the population mean of the prevalence rates of seropositivity from 2016 to 2019 is significantly different from the mean of the 2019 sample population. Hence, it is unlikely to have occurred by chance.

## Results

The total number of healthy Jordanians who donated blood or platelets at KHCC blood bank from the years 2014 to 2019 was 88,565 donors while the number of donors at all other national blood banks was 1,224,933 donors during the same study period. All individuals were adults whose age ranged from 18 to 60 years and were randomly distributed among both genders.

**Fig. 1:**
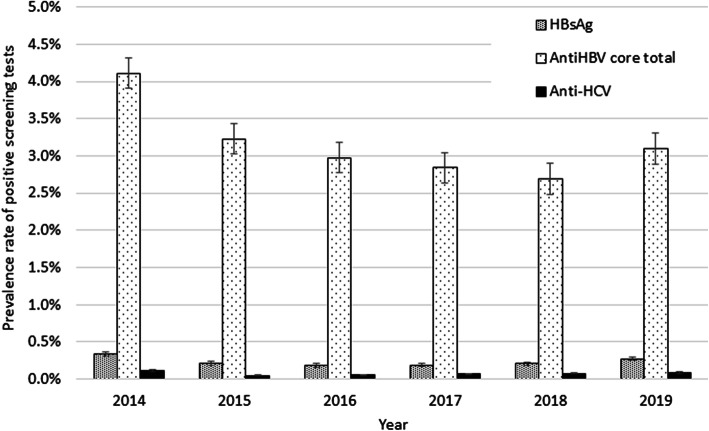
Percent Prevalence of HBsAg; Anti-HBV core total; Anti-HCV among KHCC blood Bank Donors (error bars represent standard error)

Figure 1 demonstrates the prevalence rates of HBsAg seropositivity among KHCC donors from 2014 to 2019. The prevalence rate was 0.3386, 0.2108, 0.1801, 0.1898, 0.2068, 0.2741% respectively, while the prevalence rates of anti-HBV core total antibodies were 4.1112, 3.2271, 2.9748, 2.8405, 2.6879, 3.0986% respectively. The prevalence rates of anti-HCV during the study period at KHCC was 0.1129, 0.0486, 0.0548, 0.0654, 0.0782, and 0.0839%.

The prevalence rates of the TTD among blood donors among all blood banks in Jordan were as follows: for HBsAg it was 0.46, 0.4143, 0.4013, 0.3385, 0.2929, 0.3039% between the years 2014 and 2019 respectively. While the prevalence rates of anti-HCV during the study period was 0.11, 0.1334, 0.1309, 0.1033, 0.1004, 0.0936%, respectively. As for prevalence rates of anti-HBV core total antibodies, they were available for 2019 only and it was 3.2045%. Table 1 shows the comparison between KHCC and national blood bank donors’ prevalence rates for HBsAg and anti-HCV from the years 2014 to 2019 with the associated *p*-values.

**Table 1:** KHCC and national blood bank donors-prevalence rates and *p* value for HBsAg and anti-HCV TTD markers from the year 2014–2019

TDD marker	2014	2015	2016	2017	2018	2019	*p*-value
HBsAg							
National BB	0.46%	0.41%	0.40%	0.34%	0.29%	0.30%	0.99
KHCC	0.30%	0.21%	0.18%	0.19%	0.21%	0.27%	
HCV							
National BB	0.11%	0.13%	0.12%	0.10%	0.10%	0.09%	0.99
KHCC	0.11%	0.05%	0.05%	0.07%	0.08%	0.08%	

Table 2 illustrates the numbers of positive blood bank donors for the HBsAg and anti HCV-TTD markers among KHCC (A) from the year 2009 to 2019 and national blood bank donors (B) from the year 2015 to 2019 because no raw data was available for the national blood bank donors. The prevalence rates were provided for the earlier years.

**Table 2:** KHCC and national blood bank donors-prevalence rates and *p* value for HBsAg and anti-HCV TTD markers from the year 2014–2019

A:KHCC											
TDD marker	Year										
	2009	2010	2011	2012	2013	2014	2015	2016	2017	2018	2019
HBsAg	62	79	78	63	51	42	26	23	29	37	49
AntiHBV core total	731	922	846	762	518	510	398	380	434	481	554
AntiHCV	20	26	18	28	14	14	6	7	10	14	15
**total**	**10,101**	**12,694**	**13,387**	**14,256**	**12,495**	**12,405**	**12,333**	**12,774**	**15,279**	**17,895**	**17,879**
B: National Blood Bank											
TTD marker	Year										
	2015	2016	2017	2018	2019						
HBsAg	840	782	721	672	694						
AntiHCV	269	166	200	235	211						
**total**	**208,798**	**201,426**	**220,584**	**237,112**	**230,846**						

**Fig. 2:**
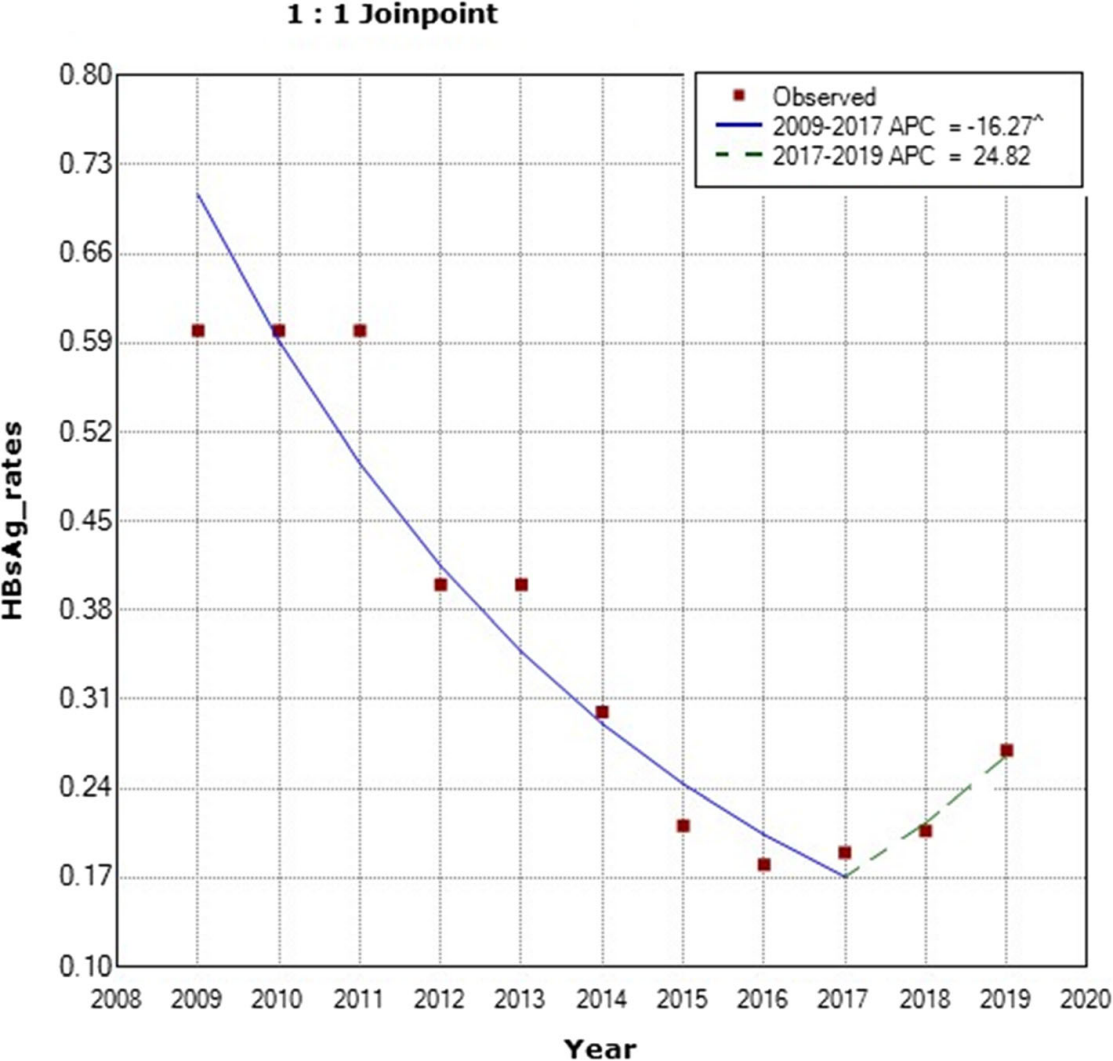
Annual Percent Change (APC) in the prevalence of HBsAg infection among healthy KHCC-blood bank donors from 2009 to 2019. (The Annual Percent Change (APC) is significantly different from zero at alpha =0.05)

We used the Joinpoint regression analysis describing the annual percent change (APC) in comparing our data from our previous publication with our current data. We found that the prevalence rate of HBsAg continued to show a significant decrease with an APC value of − 16.27 between the years 2009 to 2017 (Fig. 2). The APC was − 16.3 with a lower Confidence Level (CL) equals to − 20.6 and an upper CL equals to − 11.7 and a *p*-value of 0.0 between the years 2009–2017. While the APC was 24.8 with a lower CL of − 23 and an Upper CL equals to 102.4 and a *p*-value of 0.3 between the years 2017 to 2019 (Fig. 2).

**Fig. 3:**
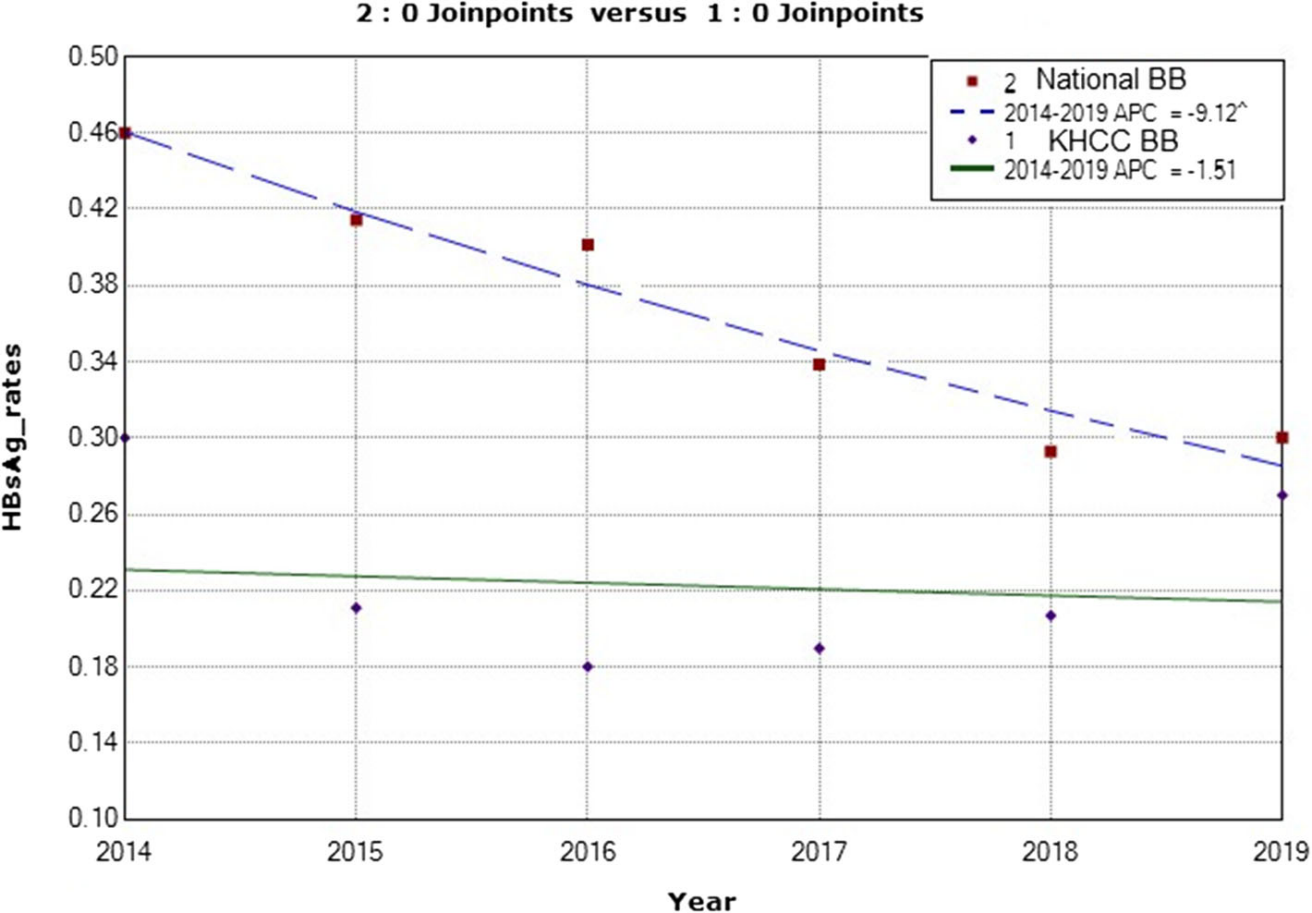
Annual Percent Change (APC) in the prevalence of HBsAg infection among healthy national blood bank donors compared to KHCC blood bank 2014 to 2019. (The Annual Percent Change (APC) is significantly different from zero at alpha =0.05)

Furthermore, there was no significant difference seen in the calculated prevalence rate among national blood bank donors compared to KHCC blood bank donors for HBsAg marker (Fig. 3). The APC was − 9.1 with a lower CL equal to − 12.2 and an upper CL equal to − 5.9 and a *p*-value of 0.0 between the years 2014 to 2019 for the national blood bank donors. For KHCC blood bank donors, the APC was − 1.5 with a lower CL of − 15.1 and an upper CL equal to 14.3 and a *p*-value of 0.8 between the years 2014 to 2019 (Fig. 3).

**Fig. 4:**
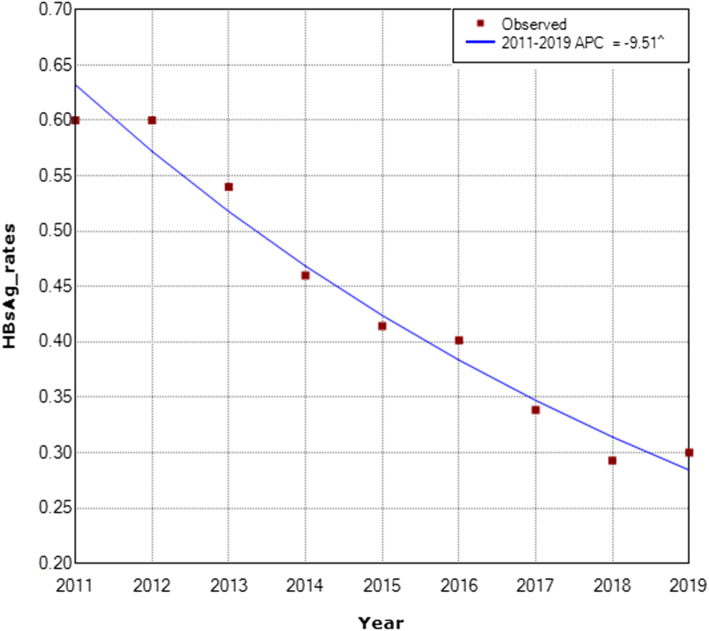
Annual Percent Change (APC) in the prevalence of HBsAg infection among healthy national blood bank donors between the years 2011 to 2019. (The Annual Percent Change (APC) is significantly different from zero at alpha =0.05)

Interestingly, there was a similar significant decrease measured in the prevalence rate of HBsAg among blood donors in Jordanian national blood banks similar to what was observed at KHCC from the years 2011 to 2019 with an APC value of − 9.51 (Fig. 4). The APC was − 9.5 with a lower CL equal to − 10.9 and an upper CL equal to − 8.1 with a *p*-value of 0.0 between the years 2011–2019 for the national blood bank donors (Fig. 4).

On the other hand, there was a slight, yet significant increase in the prevalence of HBsAg infection among KHCC-donors in 2019 using single sample Z score (*p* value is < 0.00001) observed in both KHCC and national blood bank donors (Figs. 2 and 4).

**Fig. 5:**
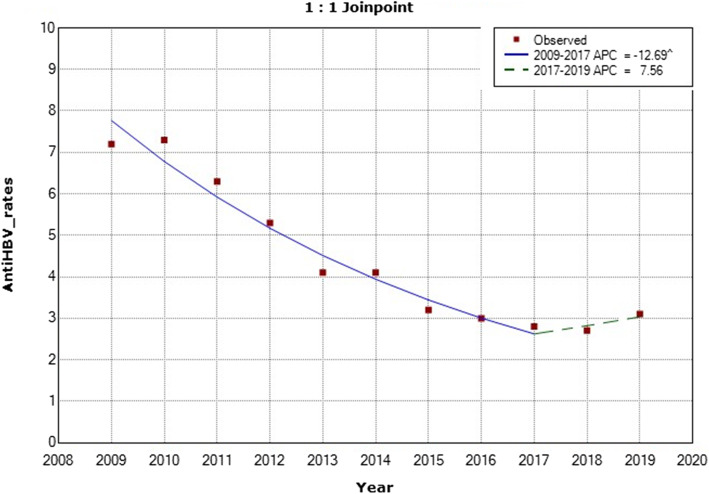
Annual Percent Change (APC) in the prevalence of anti-HBV core total antibodies among healthy KHCC-blood bank donors from 2009 to 2019. (The Annual Percent Change (APC) is significantly different from zero at alpha =0.05)

As for the anti-HBcore antibodies, our data showed that there was a continuous significant decrease in the prevalence of anti-HBV core total antibodies among healthy-KHCC blood bank donors from the year 2009 to 2017 (APC = − 12.69) (Fig. 5). However, there was a slight yet significant increase in the prevalence of anti-HBcore antibodies among KHCC-donors in 2019 using single sample Z score (*p* value is < 0.00001). No comparison was made with the Jordanian national blood bank-donors because this test was implemented in those centers in 2019; hence, there was insufficient data available for comparison.

The APC was − 12.7 with a lower CL equals to − 15 and an upper CL equal to − 10.3 and a *p*-value of 0.0 between the years 2009 to 2017. While the APC was 7.6 with a lower CL of − 16 and an upper CL equal to 37.7 and a *p*-value of 0.5 between the years 2017 to 2019 (Fig. 5).

**Fig. 6:**
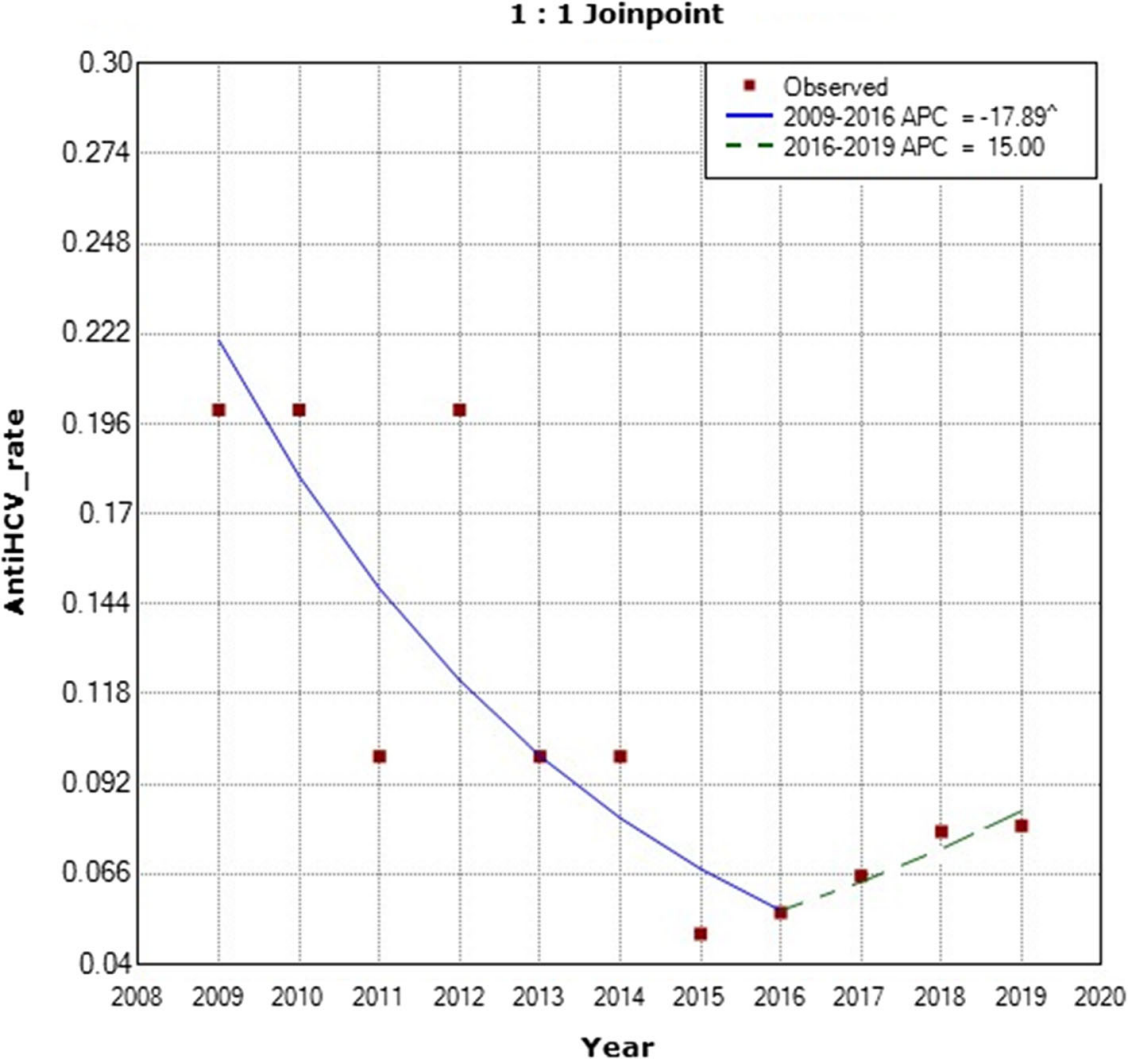
Annual Percent Change (APC) in the prevalence of anti-HCV antibodies among healthy KHCC-blood bank donors from 2009 to 2019. (The Annual Percent Change (APC) is significantly different from zero at alpha =0.05)

Furthermore, our results showed a continuous significant decrease in the prevalence of anti-HCV antibodies among healthy-KHCC blood bank donors from the years 2009 to 2016 (APC = − 17.89) (Fig. 6). The APC was − 17.9 with a lower CL equal to − 28.8 and an upper CL equal to − 5.3 with the *p*-value of 0.0 between the years 2009 and 2016. For the years 2016 to 2019, the APC was 15 with a lower CL of − 32.5 and an upper CL equal to 96.1 and a *p*-value of 0.5 (Fig. 6).

On the other hand, there was a minor-significant increase in the prevalence of anti-HCV antibodies among KHCC-donors in 2019 using single sample Z score (*p* value is < 0.00001) (Fig. 6).

**Fig. 7:**
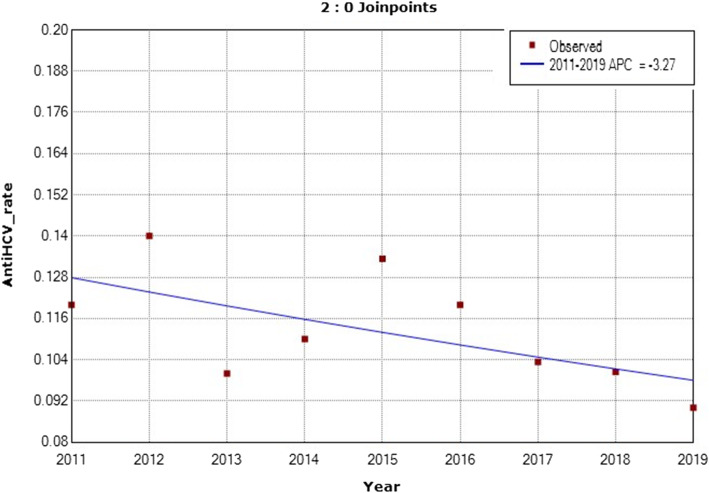
Annual Percent Change (APC) in the prevalence of anti-HCV antibodies among healthy National-blood bank donors from 2011 to 2019. (The Annual Percent Change (APC) is significantly different from zero at alpha =0.05)

The same pattern was observed among Jordanian-donors from national blood banks around Jordan where there was a significant decrease in the prevalence of anti-HCV antibodies observed among healthy donors from the year 2011 to 2019 (APC = − 3.27) (Fig. 7). The APC was − 3.3 with a lower CL equals to − 6.8 and an upper CL equals to 0.4 and a *p*-value of 0.1 between the years 2011 to 2019 (Fig. 7).

## Discussion

Our data demonstrate for the first time a significant steady decrease in the prevalence rates of HBsAg positivity among all Jordanian blood bank donors between the years 2009 to 2017. Furthermore, we show that there was no significant differences in the prevalence rates of HBsAg and anti-HCV seropositivity in donated blood as a proxy for active hepatitis B and C infections, respectively, among donors at KHCC blood bank compared with other donors at national blood bank branches in Jordan.

In addition, our results still support our previous finding that seroprevalence rate of HBsAg in Jordan is the lowest compared to neighboring countries. For example, a study performed in Duhok, Iraq, where the researchers show that the prevalence rate was 1.14% for HBsAg positive (HBsAg and anti-HBcore positive) and 8.2% for HBcore positive in 2018 compared to 0.2068 and 2.6879% respectively in Jordan [15]. Similarly in the Kingdom of Saudi Arabia (KSA), the estimation for HBsAg prevalence in 2019 was around 1.3% compared to 0.2741% for the same year in Jordan [16]. In Lebanon on the other hand; the prevalence rate for HBsAg among adults was 1.6% in a study published in 2007 [17]. The prevalence rates in Egypt were 1.22% for HBsAg in 2014 [18]. Recent studies showed that the HBsAg pooled prevalence in blood donors of both EMRO and Middle Eastern countries was 2.03% Furthermore, the prevalence rate in the EMRO countries was 1.99% while the prevalence rate was 1.62% in Middle Eastern countries. The prevalence among blood donors with more than one study was 0.24% in Iran, 2.35% in Pakistan, and 2.1% in Yemen [1].

Data concerning the prevalence of seropositive donated blood for anti-HBV core antibodies is scant, because this test is not a mandatory screening test at the majority of blood banks around the world. Nevertheless, a recent study from Saudi Arabia showed that 9.81% of blood donors at King Khalid General Hospital in Majmaah, Saudi Arabia, were positive for anti-HBc in 2018 [19] compared to 3.0986% in Jordan.

Moreover, a recent study by Bahat et al. on the prevalence of sexually transmitted diseases among Syrian immigrant pregnant women showed that the rates of HBsAg, anti-HCV, and anti-HIV seropositivity were 1.1, 0.1, and 0.03%, respectively, between the years 2012 and 2018. Additionally, Turkish women had seropositivity rates of 1.8, 0.2, and 0.08% for HBsAg, anti-HCV, and anti-HIV, respectively [20]**.**.

The significant decrease in the prevalence rates of HBsAg and anti-HBcore antibodies among blood bank donors in Jordan may be related to the mandatory infant vaccination plan which was started in 1995, and perhaps to other factors such as increased awareness and practices of proper hygiene [1, 21]. The effective protective role of anti-HBV vaccination has been documented in several recent publications. Vittal and Ghany demonstrated that vaccination regimens that followed the WHO guidelines have proven to be 90 to 95% effective in preventing HBV infection and transmission [21]. Data from Qatar has also shown a marked decline in HBV infection rates after vaccination strategies [22].

Prevalence rates of seropositive donated blood at KHCC Blood Bank for anti-HCV in Jordan showed significant steady decline in the years 2009 to 2016 similar to the decline in prevalence rates for HBsAg and anti-HBcore antibody seropositivity (APC = − 17.89) over the same period. Similar observations were made in seropositivty rates for these viral markers among national blood bank donors (APC = − 3.27). Furthermore, rates of HCV infection showed a significant decline as well. These findings may be explained, at least in part, by improved socioeconomic status and health awareness among Jordanians [23].

With respect to anti-HCV seropositivity in donated blood, we found that Jordan has relatively much lower prevalence rates to the rates in neighboring countries. Rates of seropositivity for HCV vary between countries and different regions in the same country. The Aseer region in southwestern Saudi Arabia had an anti-HCV prevalence rate of 2.2% [24], while Alexandria, Egypt, had a high prevalence rate of 23.2% [25]. In contrast, the prevalence of anti-HCV rate decreased in voluntary donors in Cairo from 4.2 to 1.5% [18]. In comparison, prevalence rates for anti-HCV were 2.61% in Pakistan [26], 2.32% in Mali [27], 3.6% in Calabar, Nigeria [28], and 0.46% in Brazil [29].

Interestingly, our data showed a small, yet a statistically significant, increase in the prevalence of HBsAg, Anti-HBcore and Anti-HCV antibodies among blood bank donors in Jordan in the year 2019 compared to the years 2016 to 2018 using the one sample z-test for mean analysis with a *p* < 0.00001. The reason for this increase in prevalence rates of hepatitis B and C virus seropositivity in 2019 is very interesting and needs a comprehensive and thorough investigation.

While our study showed for the first time the effectiveness of Jordan’s national mandatory HBV vaccination campaign in reducing HBV infection rates among Jordanian citizens until 2018, the recent increase in HBV infection rates among Jordanian citizens suggests a mandatory fast revision of the current national vaccination program implemented by the Ministry of Health. For example, we recommend the implementation of HBV screening programs for Jordanian youths and a serious and rapid revision of the public healthcare measures as well as revision of the national vaccination program including consideration for an HBV booster immunization for Jordanian adolescents to boost their immunity against HBV infection.

Several studies demonstrate the efficacy of infant Hepatitis B vaccination, yet they emphasize the need for a booster shot given to adolescents due to insufficient levels of protective HBV antibodies among these adolescents [30–33]. Furthermore, during the World Hepatitis Summit 2017 and on “World Hepatitis Day 2019” the WHO focused on the theme “Invest in eliminating hepatitis” to highlight the need for increased domestic and international funding to scale up hepatitis prevention, testing, and treatment services, in order to achieve a complete elimination of HBV infections in 2030 [34]. Based on our study, we recommend a greater public health response to HBV infections, such as screening adolescents and young adults for immunity to HBV, followed by administering a booster shot to individuals who are lacking such immunity.

## Data Availability

The datasets used and/or analyzed during the current study are available from the corresponding author on reasonable request.

## Abbreviations

HBV: Hepatitis B virus
HCV: Hepatitis C virus
TTD: Transfusion Transmitted Diseases
HBsAg: Hepatitis B surface antigen
anti- HBc: Hepatitis B core total antibodies
ELISA: Enzyme-Linked ImmunoSorbent Assay
KHCC: King Hussein Cancer Center
APC: Annual Percent Change
